# Preliminary Characterization of a Novel Aerosol Jet-Printed Strain Sensor for Feasibility Assessment in a Variable Stiffness Arterial Simulator Application

**DOI:** 10.3390/s24237725

**Published:** 2024-12-03

**Authors:** Federico Filippi, Giorgia Fiori, Annalisa Genovesi, Massimiliano Barletta, Matteo Lancini, Mauro Serpelloni, Andrea Scorza, Salvatore Andrea Sciuto

**Affiliations:** 1 Department of Industrial, Electronic and Mechanical Engineering, University of Roma Tre, 00146 Rome, Italy; federico.filippi@uniroma3.it (F.F.); giorgia.fiori@uniroma3.it (G.F.); andrea.scorza@uniroma3.it (A.S.); 2 Department of Medical and Surgical Specialties, Radiological Sciences, and Public Health, University of Brescia, 25121 Brescia, Italy; 3 Department of Information Engineering, University of Brescia, 25123 Brescia, Italy

**Keywords:** aerosol jet printing, strain sensors, strain measurements, characterization, dynamic test, arterial simulator

## Abstract

Wearable strain sensors are widespread in many fields, including the biomedical field where they are used for their stretchability and ability to be applied to non-regular surfaces. The study of the propagation speed of the pressure wave generated by the heartbeat within vessels, i.e., the Pulse Wave Velocity (PWV), is of significant relevance in this field to assess arterial stiffness, a parameter commonly used for the early diagnosis of cardiovascular diseases. In this context, arterial simulators are useful tools to study the relationship between the PWV and other hemodynamic quantities in vitro. This study aims to characterize novel strain sensors to assess their suitability within an arterial simulator capable of varying the stiffness of an arterial surrogate by varying the transmural pressure. Six sensors deposited on arterial surrogates by Aerosol Jet Printing technology were subjected to deformation through a load frame. The results show that the sensors were able to distinguish strains of 0.1%, the maximum strain was around 6–8%, and the fatigue strength depended strongly on the strain rate.

## 1. Introduction

In recent years, the use of flexible strain sensors has spread to several applications, including but not limited to structural health monitoring, aerospace, robotics, human health monitoring, and wearable sensing technology [1]. This widespread adoption was due to their various advantages, such as a wide range of deformations, a high level of wearability, and their applicability on irregular surfaces, lightweight construction, and low production cost [2]. These sensors can be classified based on the working principle by which they convert the strain into an electrical signal [3]: (a) capacitive sensors detect subtle deformations, consume little energy, and are relatively insensitive to environmental factors such as temperature and humidity; (b) piezoelectric/triboelectric sensors, often characterized by a simple and lightweight structure, do not require an external power source since they convert mechanical energy directly into electrostatic energy; (c) inductive sensors, suitable for wireless applications (e.g., rotating devices), detect displacement through changes in magnetic fields and can operate reliably in high-temperature, high-pressure, and dusty environments; and (d) resistive sensors, known for their high sensitivity over a wide measurement range, are capable of detecting various types of deformation (e.g., tension, compression, bending, and torsion) and can be designed to detect three-dimensional deformations (e.g., hydrogels, sponges, and foams). Resistive sensors are also economical compared to the others due to their simple construction and fabrication process. High-strain resistive sensors are typically composed of a conductive network integrated into a flexible substrate [4]. The conductive material, i.e., filler, is often carbon-based (e.g., carbon nanotubes [5,6,7], graphene [8], and graphite [9]) or metal-based (e.g., silver nanoparticles [10,11], silver nanowires [12], and gold meshes [13]). The substrate, i.e., matrix, is made from elastomers, such as polydimethylsiloxane (PDMS) [14], thermoplastic polyurethane (TPU) [15], and silicone rubber [16]. Many methods are used to combine the conductive material with the substrate [17]. Due to its simplicity, one of the most well-known techniques is direct mixing, which involves embedding the filler inside the elastomeric matrix to form a conductive network. Although this method is versatile, it often faces challenges in achieving uniform dispersion due to the poor compatibility between the filler and the matrix, which can lead to agglomeration, thereby reducing sensor performance [17]. Moreover, the high concentration of conductive material required to achieve sufficient conductivity can often decrease stretchability, making the manufacturing process more difficult [17]. A different method that overcomes the issues above involves the formation of electrically conductive segregated networks, where the filler is confined to the interfaces of the elastomeric matrix [17]. This approach has the advantage of reducing the amount of conductive material needed to achieve the same level of conductivity as the previous method, thus maintaining a high level of deformability. However, its downside lies in the greater complexity of constituting the conductive network. A further technique allows for the fabrication of thin skin-like sensors by directly transferring conductive materials onto an elastomeric substrate using deposition techniques, e.g., drop casting, spin coating, and spray coating [17]. Recently, printing methods [4,18] have been adopted for depositing conductive materials on stretchable substrates, such as screen printing, inkjet printing, and 3D printing. They are used to build reproducible and complex conductive patterns quickly and on a large scale.

In the context of the direct-write approach, Aerosol Jet Printing (AJP) is an emerging technology gaining attention in the field of printed electronics [19]. Aerosol Jet Printing technology offers several advantages [19,20,21]: non-contact deposition allows printing on various materials and non-planar surfaces. The high printing resolution (less than 10 µm) enables the deposition of sensors with complex geometries confined to very small spaces. Direct deposition on the substrate makes the technology cost-effective, as no production waste is generated [19], and no intermediate substances are required. In addition, the high production speed allows for large-scale process scalability. These advantages also attract significant interest in AJP technology in the biomedical field [19,22,23]. Motion analysis (e.g., knee, wrist and hip movement, and finger gripping) requires the development of highly flexible sensors that can adapt to uneven surfaces [3,4]. The measurement and monitoring of vital parameters (e.g., body temperature, heart rate, breath detection [24], and blood pressure [25]), necessitate the development of thin and highly sensitive sensors [4]. In clinical practice, arterial stiffness is another significant indicator, as it reflects the health of the cardiovascular system and can reveal the presence of cardiovascular diseases (CVDs) at an early stage, before the onset of typical symptoms [26]. In this context, the Pulse Wave Velocity (PWV) is one of the most commonly used parameters to estimate arterial stiffness. It is typically measured by estimating the Pulse Transit Time (PTT), defined as the time difference between the instants at which the pressure wave produced by the heartbeat travels between two different sites in the arterial system [27]. In this scenario, arterial simulators are valuable tools for studying the relationship between the PWV and other hemodynamic variables [28], as they provide the opportunity to simulate the phenomenon of interest in a reproducible manner, isolating it from the many factors that contribute to its variability in the human body. In recent scientific literature, a prototype arterial simulator has been proposed [29] that can reproduce a variation of the PWV within a silicone rubber surrogate by varying its stress–strain state through the difference between the internal and external pressure on its walls. In [29], the transit of the pressure wave within the conduit was detected using common electrical resistance strain gauges glued directly to the tube. However, the limit of the application is a reduced possible transmural pressure range, as the strain gauges were significantly stiffer than the rubber vessel (i.e., by about three orders of magnitude) and could potentially introduce a significant insertion error. Moreover, since the arterial surrogate is enclosed inside a container and placed under pressure, it would be difficult to use non-contact-based methods. For these reasons, the novelty of the present study is to investigate alternative measurement solutions for the application in [29], specifically proceeding with the preliminary characterization of an innovative strain transducer and evaluating its applicability to the setup described in [29]. The proposed transducer is a particular stretchable strain sensor for wearable electronics applications composed of a carbon-based conductive powder and a polymer matrix. It was directly deposited on the arterial surrogate using AJP technology. The main goal is to evaluate its suitability with the application, also in terms of the minimum and maximum detectable strain in accordance with the strain range affecting the arterial surrogate.

The study is organized as follows: Section 2 addresses the application requirements based on the tests conducted in [29]. Section 3 describes the materials used for the characterization, including the specimens under test and the measurement chain. Section 4 provides details of the measurement campaign carried out. In Section 5, the indices used for the characterization are introduced and described. Section 6 deals with the measurement uncertainty evaluation. In Section 7, the results obtained from the measurements are presented and discussed. Finally, in Section 8, both the suitability of the tested sensors with the application and possible future developments are outlined.

## 2. Application Requirements

The working principle of the arterial simulator, as described in [29], relies on varying the difference between the internal and external pressure (i.e., transmural pressure) applied to the walls of the arterial surrogate to alter its stress–strain state. By utilizing an elastomeric conduit, for which the elastic modulus is dependent on the strain state [30], its stiffness can be modified. As a result, a variation in the Pulse Wave Velocity is produced according to the Moens–Korteweg equation [31]:

(1) $$PWV=\sqrt{\frac{Eh}{\rho d}}$$

where *E*, *h*, and *d* are the Young modulus, the thickness, and the inner diameter of the surrogate, respectively, while *ρ* is the density of the inner fluid.

The PWV can be derived by measuring the time the arterial pulse takes to travel a well-defined distance [29]. The transit of the wave is detected by measuring the circumferential strain of the conduit caused by the pressure pulse. Therefore, the sensors on the outer wall of the arterial surrogate will be subject not only to the deformation caused by the pressure pulse, but also to that caused by the application of transmural pressure. In this study, we intended to evaluate the suitability of the sensors only with the latter. Therefore, the first task is to estimate the circumferential strain undergone by the conduit to determine the strain range to which the sensors should be subjected. Referring to a cylindrical coordinate system, the application of a transmural pressure generates a plane stress condition. In particular, the stresses imposed by the transmural pressure can be described by Lamé’s equations [32]:

(2) $$\sigma_{t}=\frac{p_{i}r_{i}^{2}-p_{o}r_{o}^{2}}{r_{o}^{2}-r_{i}^{2}}+\frac{r_{i}^{2}r_{o}^{2}(p_{i}-p_{o})}{r^{2}(r_{o}^{2}-r_{i}^{2})}$$

(3) $$\sigma_{r}=\frac{p_{i}r_{i}^{2}-p_{o}r_{o}^{2}}{r_{o}^{2}-r_{i}^{2}}-\frac{r_{i}^{2}r_{o}^{2}(p_{i}-p_{o})}{r^{2}(r_{o}^{2}-r_{i}^{2})}$$

where *σ_t_* and *σ_r_* represent the tangential and radial stress of the conduit walls, respectively, as a function of the radius *r*. Specifically, *r_i_* and *r_o_* are the internal and external radius of the conduit, while *p_i_* and *p_o_* represent the internal and external pressure on the walls, respectively. On the assumption that the material constituting the surrogate is isotropic, the stress state can be related to the strain state using the following constitutive equations [32]:

(4) $$\varepsilon_{t}=\frac{1}{E}(\sigma_{t}-\nu \sigma_{a}-\nu \sigma_{r})$$

(5) $$\varepsilon_{r}=\frac{1}{E}(\sigma_{r}-\nu \sigma_{a}-\nu \sigma_{t})$$

(6) $$\varepsilon_{a}=\frac{1}{E}(\sigma_{a}-\nu \sigma_{r}-\nu \sigma_{t})$$

where *ε_t_*, *ε_r_*, and *ε_a_* are the tangential, radial, and axial strains, respectively, and *ν* is the Poisson’s ratio of the material. In addition, assuming that the tube is not pre-tensioned and that its ends are open, the axial stress can be considered negligible (*σ_a_* = 0) [32].

### 2.1. Maximum Detectable Strain

For the estimation of the maximum tangential strain to be detected by the sensors, Equations (2)–(4) can be used. Referring to [29] and the above discussion, an external pressure set at *p_o_* = 50 kPa and a maximum internal pressure *p_i_* = 75 kPa were considered. Therefore, a maximum transmural pressure value ∆*P_max_* = 25 kPa was assumed. This value, applied on a tube with inner radius *r_i_* = 7 mm, outer radius *r_o_* = 9 mm, and Young modulus *E* = 3.5 N/mm^2^, carried out a stress–strain condition on the outer perimeter of the conduit of the following:

*σ_t_* ≈ 6 × 10^3^ Pa (7)

*ε_t,max_* ≈ 14 × 10^−3^ mm/mm ≈ 1.4% (8)

### 2.2. Minimum Detectable Strain

According to [29], the transmural pressure is changed by the operator in a fixed step of ∆P_min_ = 5 kPa. Consequently, the minimum detectable strain for the present application was expected to distinguish different strain states as a function of ∆P_min_. Keeping constant *p_o_*, as mentioned in Section 2.1, applying Equations (2)–(4), it followed that the minimum strain undergone by the conduit in the tangential direction corresponded to the following:

*ε_t,min_* ≈ 3 × 10^−3^ mm/mm ≈ 0.3% (9)

## 3. Materials

This section describes the materials and instruments used for the characterization of the AJP-based strain sensors. Specifically, the configuration of the specimens under test, which consist of a rubber substrate (Section 3.1) and a conductive paste deposited on top (Section 3.2) is shown. Moreover, the load frame used to apply tensile strain to the specimens (Section 3.3), and the components of the measurement chain for acquiring strain and resistance data (Section 3.4) are described.

### 3.1. The Arterial Surrogates

Three arterial surrogates were used as a substrate for the specimens, obtained from the same tube. They were made of silicone rubber, with an inner diameter of 14 mm and a wall thickness of 2 mm. The supplier indicates a hardness of 60 Shore A, which corresponds approximately to an elastic modulus of 3.5 MPa, according to [33]. The material selected for the arterial surrogate was chosen due to its close similarity to the one used in [29]. The maximum operating temperature is 180 °C. This allowed us to perform the after-printing sensors’ curing, avoiding unexpected changes in mechanical properties of the silicon rubber. Each surrogate had a length *L_t_* = 120 mm in the axial direction. This specific size allowed us to easily fit the specimens inside the load frame.

### 3.2. AJP-Based Sensors

The transducers investigated in this study consisted of a stretchable carbon conductive paste (DuPont Intexar PE671, DuPont Ltd., Bristol, UK) that exhibits a piezoresistive behavior as the strain varies. Their main geometrical and resistive characteristics are listed in Table 1. Although the manufacturer does not provide specific information on the elastic modulus of the material, it recommends the application to thermally stable substrates for wearable electronics applications.

**Figure 1:**
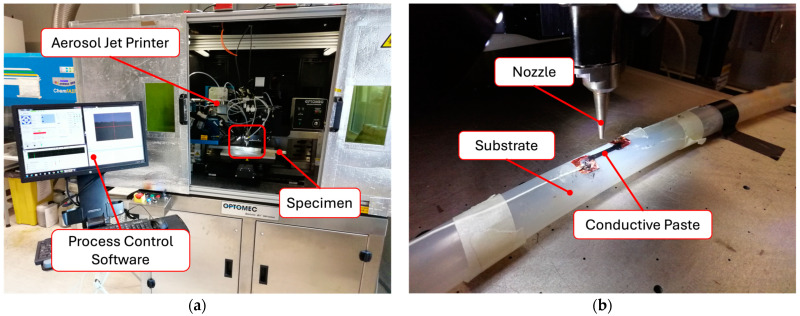
Overview of the Aerosol Jet Printing process: (**a**) the equipment used for sensor printing, the red box indicates the printing area and (**b**) an example of a tube undergoing the printing process.

The paste was deposited directly on the external surface of the arterial surrogates using the AJP technique (Figure 1). The printing process involved alternating the deposition of multiple conductive paste layers with curing in an oven (130 °C for 15 min), repeated three times. The pneumatic configuration with the wide deposition head and a deposition speed of 1 mm/s was used for printing. The flow rate set was 2000 SCCM (@1.23 psi), 2000 SCCM (@24.6 psi) and 1000 SCCM (@0.72 psi) for the three deposition phases of the conductive paste, respectively. In Figure 1a, the equipment used for sensor printing is shown, and in Figure 1b, an example of a specimen during the printing process can be observed.

**Figure 2:**
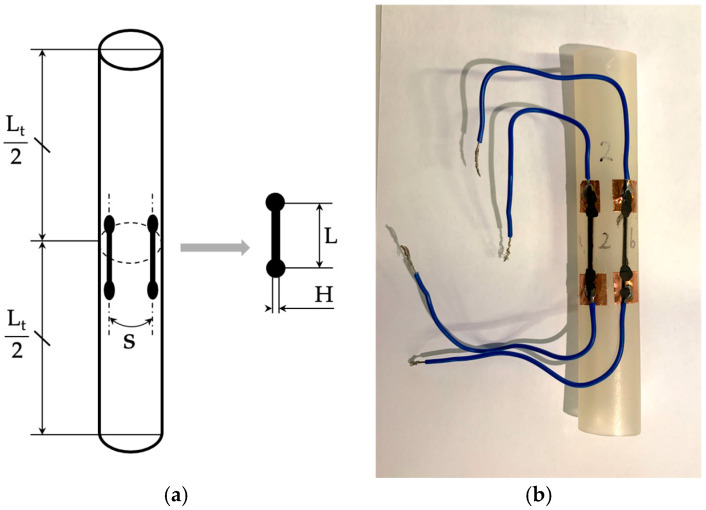
Example of a specimen after the production process: (**a**) configuration of the two sensors on the silicon rubber substrate and (**b**) specimen before the measurement campaign.

For application in the arterial simulator, the circumferential strain was of primary interest (Section 2). However, for ease of application, in this case, the sensors were placed with their strain-sensitive direction parallel to the conduit’s axis (Figure 2), and their response to axial deformation was evaluated using a tensile testing machine according to the reference value found in Equation (8). A schematic representation of a specimen is shown in Figure 2a, where two sensors were deposited symmetrically with respect to the central section of the tube at a distance *S* = 12 mm. Quantities L and H represent the portion of the sensor printed directly on the rubber and the width of the strain-sensitive element, respectively. The thickness of the sensor is not represented.

The sensor ends were connected to copper sheets glued to the tube, onto which the electrical wires used for resistance measurement were soldered. An example of a completed specimen is shown in Figure 2b.

Overall, three specimens were produced, with a total of six sensors. Because the curing process was performed on one silicone rubber substrate at a time, having at least two sensors on a surrogate was also useful in assessing the variability of the production process. The latter was affected by, among other factors, the different thickness of the sensors due to the different number of layers for each and the different number of curing steps performed on each silicone rubber substrate. The main characteristics of the sensors are listed in Table 1.

### 3.3. Load Frame

**Figure 3:**
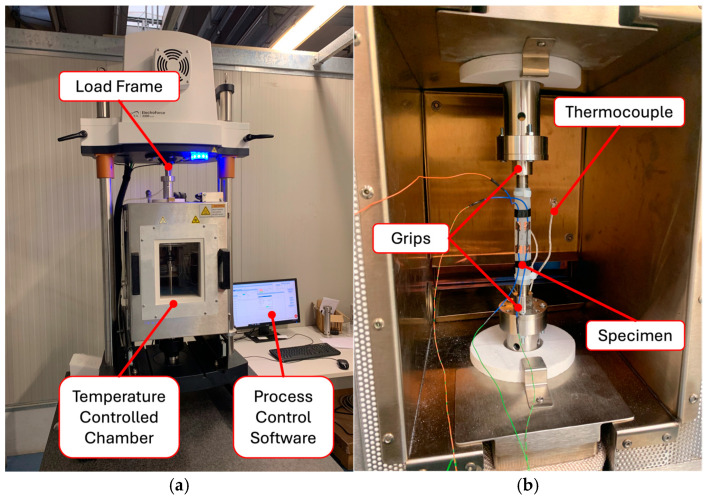
The load frame used for tensile tests on specimens: (**a**) the machine with the temperature-controlled chamber and (**b**) a detail of a specimen positioned with proper grips. The thermocouple used for temperature monitoring is also visible.

For the mechanical characterization, each specimen was positioned, using suitable grips, within a load frame (ElectroForce 3300 series) capable of performing both static and dynamic tensile tests (Figure 3). The system is equipped with a temperature-controlled chamber with a tolerance of ±0.5 °C (Figure 3a). The temperature is monitored by a type K thermocouple. The initial distance between the grips *L*_0_, which determines the length of the specimens in the absence of deformation, was set at 100.0 ± 0.3 mm. No pre-tensioning was applied to the specimens before testing.

Once the *L*_0_ was set, the displacement between the grips Δ*L* was monitored by a High-Aspect-ratio Dielectric Sensor (HADS) installed directly on the machine. Figure 3b shows a detailed view of a specimen mounted within the load frame.

### 3.4. Measurement Chain

**Figure 4:**
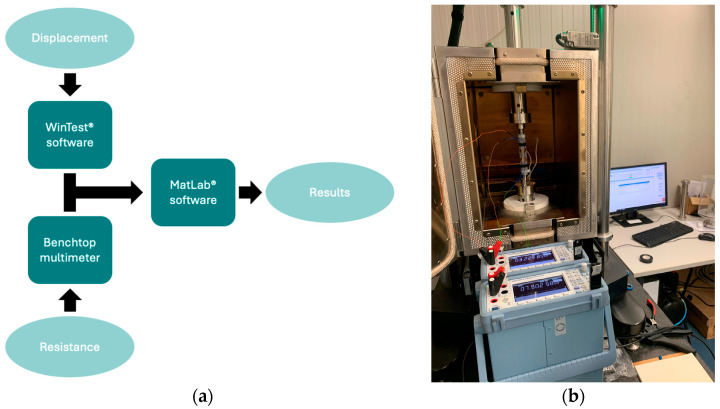
The measurement chain used for signal acquisition: (**a**) block diagram of the main steps of the acquisition process and (**b**) an overview of the measurement chain ready to carry out the acquisition campaign.

The overall scheme of the measurement chain configuration is shown in Figure 4a. The displacement values ∆*L*, imposed on the specimens by the load frame, were recorded directly by the proprietary software of the machine (WinTest^®^, v.8.3). In addition, two-wire resistance measurements were carried out by a benchtop multimeter (Yokogawa DM7560, Yokogawa Europe B.V., Amersfoort, The Netherlands) to collect resistance values simultaneously from both AJP-based sensors. The signals were acquired on USB memory in two separate files (.csv) and then imported into MatLab^®^ (v. 9.14.0) for post-processing operations. Figure 4b provides an overview of the measurement chain used for data acquisition.

## 4. Measurement Campaign

This section describes some details of the mechanical tests carried out on the specimens in order to characterize their performance and thus evaluate their suitability with the application in [29]. As mentioned above, the specimens were subjected to tensile tests by imposing known strain values. Since both the behavior of the sensors and their tensile limit before failure were not known, a strain range of 50% of the maximum required by the application (Section 2) was chosen to perform the tests as a precaution. Consequently, the maximum deformation value applied during the tests was *ε_test,max_* = 0.7%.

The displacement and resistance signals were acquired using the equipment described in Section 3.4, both at a sampling frequency of 20 Hz. Each test lasted 5·10^3^ s, during which 10^5^ samples were collected (approximately 83 min). A waiting time of 120 min was observed before starting the tests to allow the measurement chain warmup. The acquisitions of displacements and resistances started simultaneously, synchronized via a trigger event. Since the intended application of the sensors does not involve a significant change in system temperature, the dependence of resistance as a function of temperature was not evaluated in this study. For this reason, all tests were conducted under repeatable conditions, maintaining a constant temperature of 24.0 ± 0.5 °C. Further details specific to each test performed are provided below.

### 4.1. Static Test

The static test involved subjecting the specimen to strain steps ∆*ε* = 0.1%, from the undeformed state, i.e., *ε***_0_** = 0, to *ε_test,max_*. Each imposed deformation interval was applied by the load frame in 100 ms and maintained constant for 600 s. Despite the application of a constant strain, the resistance value *R* was expected to change over time because of the viscoelastic behavior due to the polymeric nature of the specimens [34,35]. Therefore, *R* was estimated as the mean ± standard deviation (SD) of the last 20 values acquired at the end of 600 s, just before the next strain step ∆*ε* was applied. Conversely, the initial resistance *R***_0_** was assumed as the mean ± SD of the last 20 values acquired at the beginning of the test, just before the first strain step was imposed. With the aim to estimate the uncertainty contribution due to the hysteresis phenomenon, a load–unload cycle was performed.

### 4.2. Dynamic Test

The dynamic test involved subjecting the specimen to varying deformation values by imposing a sine function through the load frame. Specifically, two tests were performed for each specimen, in which the sine wave frequency was set to 0.01 and 0.1 Hz. The deformation range remained as specified in the static test above, starting from *ε***_0_** and to *ε_test,max_*. Considering that each period of the sine wave corresponded to one load–unload cycle, 45 and 450 load–unload cycles were performed for the first and second tests, respectively. The resistance value was expected to settle as a function of strain as the cycles increased. Consequently, the resistances *R*_0_ and *R* for the dynamic test were estimated and expressed in terms of the mean value ± SD from the load–unload cycles once the settling period had passed. Thus, it was possible to assess the contribution of uncertainty due to the repeatability of the measurements.

### 4.3. Maximum Elongation Test

The maximum elongation test involved subjecting the specimen to varying deformation values with a ramp pattern, up to the point of the sensor breaking, i.e., when it no longer provided electrical continuity. For this test only, the load frame was set to impose linearly increasing strain values from *ε***_0_** to 10.0% with a rate of 1 mm/min. The maximum strain set for this test is not related to the requirements of the application, as in Equation (8), but was set to evaluate the maximum elongation that the sensors could reach. The maximum elongation test was applied to one specimen, and thus to two AJP-based sensors.

### 4.4. Fatigue Strength Test

The fatigue strength test is applied by subjecting the specimen to a cyclic deformation range of 10^4^ cycles. Specifically, the sensors underwent deformations between *ε*_0_ and *ε_test,max_*. The test, performed on a single specimen, was conducted at a frequency of 1 Hz.

## 5. Characterization Indices

The characterization indices are parameters used to assess whether the sensors investigated in this study meet the application requirements described in Section 2.

The first purpose was to determine the minimum detectable strain (MinDS) by the sensors. In order to distinguish two strain values, *ε_i_* and *ε_j_*, that correspond to two resistance values provided by the sensor, *R_i_* and *R_j_*, with uncertainty contributions, *σ_Ri_* and *σ_Rj_*, the following inequality must be satisfied [36]:

(10) $$|R_{i}-R_{j}|>\sigma_{Ri}+\sigma_{Rj}$$

Assuming that the change in strain is unitary, i.e., ∆*ε_ij_* = |*ε_j_* — *ε_i_*| = 1 mm/mm, the change in resistance ∆*R_ij_* = |*R_j_* — *R_i_*| is an expression of sensitivity, as follows:

(11) $$sensitivity=\;\frac{{∆R}_{ij}}{{∆\varepsilon }_{ij}}$$

Therefore, it is necessary to estimate both the sensitivity and accuracy, in terms of measurement uncertainties, to determine the minimum strain variation that the sensor under test can distinguish (MinDS). The same reasoning applies if the resistance R is replaced by the relative resistance variation with respect to the initial value, i.e., ∆*R*/*R*_0_, in Equations (10) and (11). In this case, Equation (11) becomes an expression of the gauge factor (*GF*), as follows:

(12) $$GF=\;\frac{\frac{∆R}{R_{0}}}{\varepsilon }$$

Consequently, to assess the MinDS of the AJP-based sensors, it was necessary to define and estimate the two indices mentioned above, i.e., the *GF* and the measurement uncertainty *σ*_∆*R*/*R*0_. The methods by which these two indices were obtained are described in Section 5.1 and Section 6.3.

As a second step, the maximum strain detectable by the sensors, i.e., the maximum detectable strain (MaxDS), was investigated. This parameter can be quantified through two indices, i.e., the stretchability and fatigue strength, which provide indications of the strain range of the sensors. Details on their assessment are provided in Section 5.2 and Section 5.3.

### 5.1. Gauge Factor

The gauge factor is a key parameter, as it indicates the sensitivity of the transducer [4]. A high sensitivity allows for an easier detection of subtle deformations. In this regard, the linear behavior of the transfer function is another desirable characteristic in the production of this type of sensor.

The GF was estimated as the slope of the straight line obtained by applying a weighted least squares linear regression to the acquired resistance values as a function of the strain. The variance of the resistance measurements from the load–unload cycles was estimated for each strain value and the inverse of each was used in the weighted regression. The coefficient of determination R^2^ was assessed to quantify the goodness-of-fit. The gauge factor was computed for both static and dynamic tests.

### 5.2. Stretchability

The stretchability is a further key factor that characterizes a stretchable sensor [4]. It depends, among other factors, on the type of conductive material, the properties of the material that constitutes the matrix, and the amount of filler in the matrix. In general, a sensor with a higher stretchability finds use in more applications. In this study, the stretchability was estimated in terms of the percentage strain at which the sensor no longer provided electrical continuity, through the maximum elongation test.

### 5.3. Fatigue Strength

The fatigue strength denotes the ability to maintain structural integrity, i.e., to not undergo significant alteration in response to a stimulus when subjected to cyclic loading. In this study, the sensor resistance within the imposed strain range during the dynamic tests was evaluated and expressed in terms of the number of cycles through the fatigue strength test.

## 6. Measurement Uncertainty Analysis

This section outlines the procedure adopted for estimating the measurement uncertainty, expressed as the standard deviation, of the quantities used to calculate the indices in Section 5. First, the uncertainty of the directly measurable quantities, i.e., displacement ∆*L* and resistance *R*, was estimated. Then, by properly combining all the contributions, the uncertainty of the strain ε and relative resistance variation ∆*R*/*R*_0_ was estimated.

### 6.1. Displacement and Strain Uncertainties

As mentioned above, the *L*_0_ was set at a distance of 100.0 ± 0.3 mm using a standard reference gauge block provided by the manufacturer of the load frame. The uncertainty associated with the imposed displacements ∆*L* between the grips of the load frame was estimated from the calibration certificate of the testing machine, i.e., *σ*_Δ*L*_ = 0.025 mm. By combining the relative uncertainty contributions of *L*_0_ and ∆*L*, a maximum uncertainty for *ε* of 0.03% was obtained. Since the minimum strain value applied to the specimen was 0.1%, its contribution to the estimation of uncertainties for derived quantities, e.g., the gauge factor, can reasonably be considered negligible.

### 6.2. Resistance and Relative Resistance Variation Uncertainty

The uncertainty associated with the resistances R_0_ and R, acquired with the bench multimeter, was estimated by combining the repeatability contribution, and the uncertainty contribution provided by the instrument, which also depends on the range set (i.e., 1–10 kΩ according to the sensor under test). The uncertainty of the resistance variation relative to the initial value *σ*_Δ*R*/*R*0_ was estimated by summing in quadrature the contributions from the readings of the initial and final resistance values.

### 6.3. Sensor Transfer Function and Prediction Bounds

As described above, the sensor transfer function was derived by the weighted least squares fit applied to variations in the relative resistance measurements as a function of the imposed strain. The weights were assessed as the inverse of the variance of the relative resistance variation values, combining the contributions in Section 6.2 with the repeatability contribution estimated from the load–unload cycles. Prediction bounds were estimated by considering the uncertainties of the coefficients obtained from the linear regression model. Specifically, they were assumed as the maximum interval between the two extremal lines of the pencil of lines derived from the regression coefficients and their uncertainties.

## 7. Results and Discussion

### 7.1. Static Test

**Figure 5:**
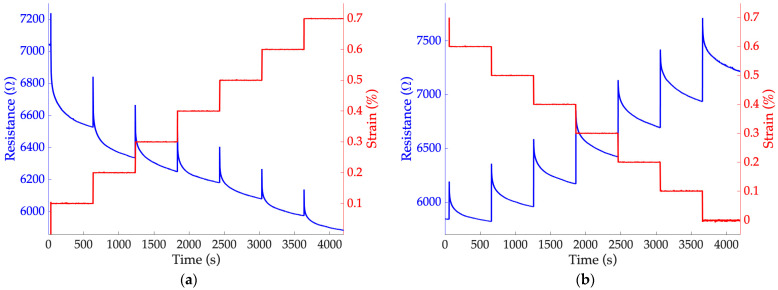
Trends of resistance over time acquired from sensor 1a during the static test: response during (**a**) the loading phase and (**b**) unloading phase.

In Figure 5, an example of the sensor response to the application of static strain steps can be observed, both in the loading and unloading phases (Figure 5a and Figure 5b, respectively). It can be noted that during the application of the steps ∆*ε*, there is always an increase in resistance. Then, when the input strain remains constant, the resistance decreases, following an exponential law. This behavior is known in the literature as ‘overshoot’ [37], and its magnitude is directly proportional to the strain value and strain rate for the same material [38]. This phenomenon highlights the viscoelastic nature of the specimens [34,35].

Overall, considering the response of the sensor throughout the static test, it can be observed that the resistance value decreases with increasing strain (Figure 5a), and vice versa (Figure 5b). This behavior, known in the literature [39], could be due to the strain of the polymer matrix, which in this case corresponds to an elongation of the sensor, allowing better contact between the conductive particles within a certain strain range [40]. Based on these considerations, it is assumed that this effect was amplified by the overlapping layer structure of which the sensors are composed (Section 3.2), promoting particle contact between the different layers. This issue deserves further investigation in the future by comparing sensors with varying number of layers.

**Figure 6:**
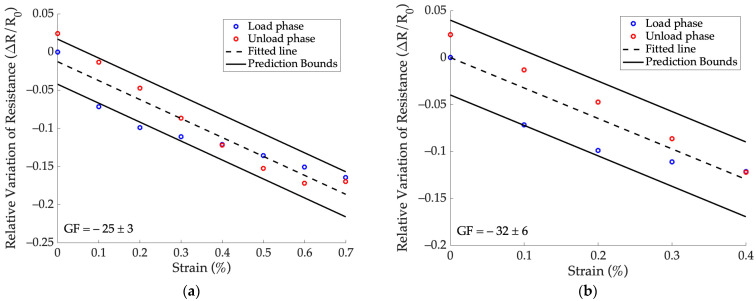
Relative resistance variation during loading and unloading phases as a function of the strain in the static test for sensor 1a. The line resulting from weighted least squares linear regression with its prediction bounds are also shown: (**a**) entire strain range and (**b**) strain range up to 0.4%.

Figure 6 shows the resistance results obtained for sensor 1a for both the loading and unloading phases as a function of the imposed strain. The straight line from the linear regression and corresponding prediction bounds are also shown. In Figure 6a, the trend for the entire imposed strain range can be observed, while a focus on strain values up to 0.4% is provided in Figure 6b. Gauge factor outcomes (expressed as mean ± SD), R^2^ coefficients, prediction bounds, and the MinDS obtained for each sensor are listed in Table 2 for both strain ranges in Figure 6.

Globally, sensors on the same surrogate exhibit similar behavior, while the wide prediction bounds confirm a hysteresis phenomenon. It can be noticed that higher R^2^ values are obtained for the strain range up to 0.4% and the response seems more linear. In this range, the minimum detectable strain of the sensors is 0.3%, while this is not always valid considering the strain interval up to 0.7%.

### 7.2. Dynamic Test

**Figure 7:**
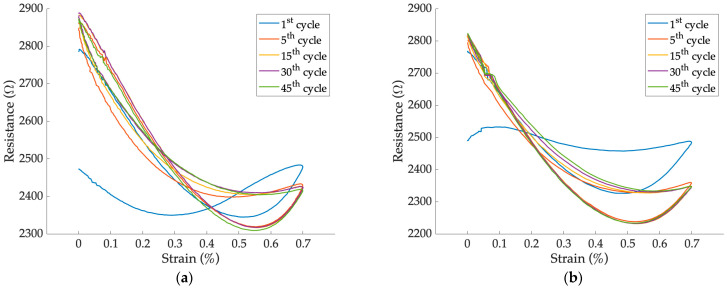
Response of sensor 2b at sinusoidal strain values: dynamic test at (**a**) 0.01 Hz and (**b**) 0.1 Hz.

Figure 7 shows the behavior of a sensor subjected to cyclic strain loading in the range of 0.0 to 0.7% at frequencies of 0.01 Hz (Figure 7a) and 0.1 Hz (Figure 7b). It can be observed that the sensor required at least ten preconditioning cycles before reaching a stable behavior.

**Figure 8:**
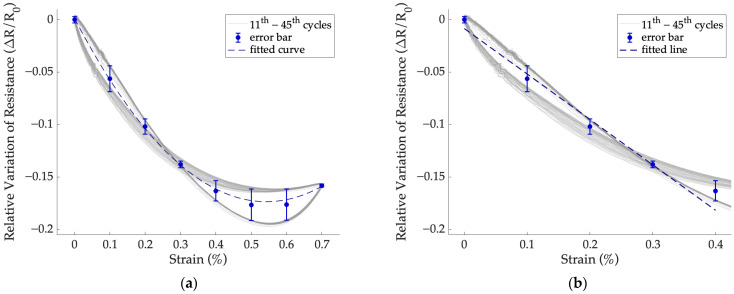
Interpolation of the response of sensor 2b as a function of the strain applied during the dynamic test at 0.01 Hz. The error bars represent the standard deviations estimated from the measurements performed during the loading–unloading phases for each strain value: (**a**) second-order polynomial fitting over the entire strain range and (**b**) linear fitting within the strain range up to 0.4%.

The change in resistance within the strain range of 0.0 to 0.7% does not appear linear. This hypothesis was confirmed by the fact that fitting the trend with a second-order polynomial (Figure 8a) yields an R^2^ coefficient > 0.99. Therefore, in this case, the weighted linear least squares regression was carried out only for strains up to 0.4% (Figure 8b).

In Table 3, the GF values calculated during the dynamic tests are shown for a strain interval of 0.0 to 0.4% at frequencies of 0.01 and 0.1 Hz, respectively. Moreover, the corresponding R^2^ values for each fitting, the prediction bounds, and the minimum detectable strain are provided.

In the applied strain range, a general increase in sensor sensitivity was found between the dynamic test at 0.1 Hz and the static test. This is shown by an increase in absolute GF values ranging from 37% to 95%. The phenomenon could be justified by the fact that by increasing the strain rate (i.e., increasing the test frequency at the same strain interval), the specimen responds more rigidly [41]. A decrease in prediction bounds between the dynamic and static tests ranging from 20% to 65% was found, suggesting a reduction in the hysteresis phenomenon. The minimum detectable strain of the sensors under dynamic test was between 0.1 and 0.2%, in agreement with the specifications required by the application.

### 7.3. Maximum Elongation Test

**Figure 9:**
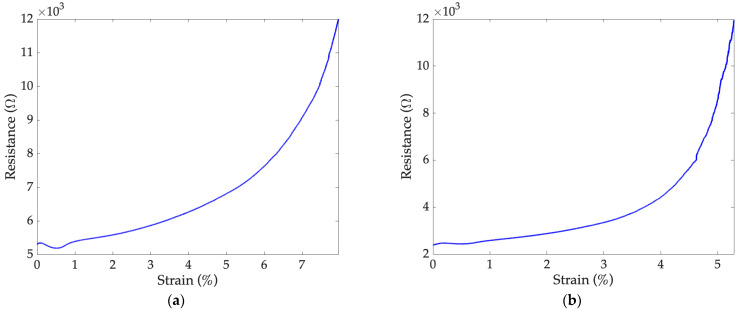
(**a**) Sensor 2a and (**b**) sensor 2b behavior during the maximum elongation test performed at 1 mm/min.

Figure 9 shows the resistance trends as a function of the changes in the strain during the maximum elongation test carried out on specimen 2. It can be observed that sensors 2a and 2b reached a maximum strain of 8.0% and 5.3%, respectively. These results are consistent with the application requirements in Equation (8). In addition, the maximum strain values obtained are in agreement with those reported in the current literature for sensors produced through AJP technology [19].

In Figure 9, the sensor response in the strain range between 0.0–1.0% is shown. Indeed, at first, the resistance increased, then decreased for a limited interval, and then increased again. This behavior, described in [40,42], depends, among other factors, on the percentage of carbon particles within the conductive paste. It would be interesting to impose a pre-tensioning on the sensors before their testing to obtain a more stable behavior of the sensors for a wider strain range.

### 7.4. Fatigue Strength Test

**Figure 10:**
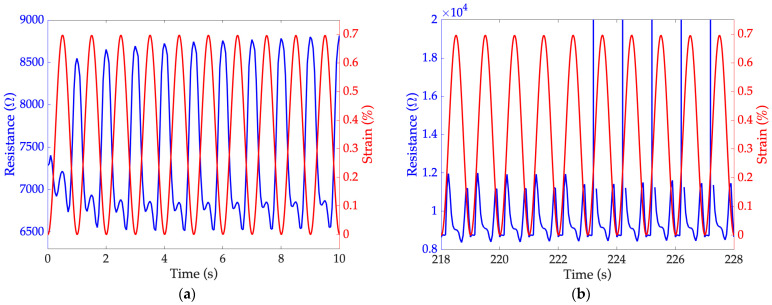
Resistance variations of sensor 1a with respect to strain values, related to fatigue strength test: (**a**) behavior of sensor still intact, at the beginning of the test and (**b**) behavior of sensor after crack formation and at the time of rupture.

Specimen 1 was subjected to the fatigue resistance test: failure occurred at the 223rd 237th cycle for sensors 1a and 1b, respectively. Figure 10 shows the response of sensor 1a as a function of the applied strain when it is still intact (Figure 10a) and after crack formation (Figure 10b). A change in sensor behavior can be observed near the failure (Figure 10b) compared to the beginning of the test (Figure 10a). Crack propagation is highlighted by a general increase in the average resistance values. Moreover, a response shift was found: while a minimum strain corresponds to a maximum resistance (Figure 10a), a minimum resistance was observed near the minimum strain. Based on these considerations, the dependence of the sensor resistance on the frequency requires further investigation, since, in the context of dynamic tests, the application of the same strain at a frequency of 0.1 Hz did not cause any failure even after 450 cycles.

## 8. Conclusions

This work presented the preliminary characterization of innovative strain sensors based on Aerosol Jet Printing technology to assess their suitability within an arterial simulator capable of varying the stiffness of an arterial surrogate through changes in transmural pressure. The sensors were expected to distinguish minimum strain values *ε_min_* = 0.3% and detect maximum strain values *ε_max_* = 1.4%, as required by the application. To conduct the characterization tests, three tubes made of silicone rubber were used as the substrate. Two sensors, composed of a piezoresistive conductive paste, were deposited directly on each substrate through AJP technology. To measure the change in resistance as a function of strain, the three specimens underwent tensile tests in the strain range of 0.0 to 0.7% using a load frame. The minimum detectable strain was determined through one static test and two dynamic tests. In the static test, constant strain steps of 0.1% were applied from the undeformed state to 0.7%, while in dynamic tests, a sinusoidal input was applied at frequencies of 0.01 and 0.1 Hz. To assess the maximum detectable strain, a maximum elongation test and fatigue strength test were carried out. The experimental results suggest that the sensors tested in this study partially meet the requirements of the application. In the future, higher types of AJP-based sensors should be tested, also including different conductive pastes and varying the number of layers. As a last remark, further studies are going to be carried out, where the constant strain is maintained for a longer time in the static test, to evaluate the viscoelastic behavior of the specimen more comprehensively. Alongside this, it would be interesting to investigate the sensor behavior when a pre-tensioning state is applied in both static and dynamic tests.

## Figures and Tables

**Table 1:** Main geometrical and resistive characteristics of each sensor.

Specimen	Sensor	Dimensions (L × H)	Nominal Resistance (kΩ)
1	a	17.5 mm × 1.3 mm	7.03
	b	17.9 mm × 1.4 mm	3.59
2	a	18.7 mm × 1.1 mm	7.50
	b	18.8 mm × 1.2 mm	3.23
3	a	15.9 mm × 1.3 mm	1.39
	b	16.2 mm × 1.3 mm	1.02

**Table 2:** Gauge factor, prediction bounds, and minimum detectable strain for each sensor estimated for two strain intervals.

Sensor	Strain Interval (%)	GF	R2	Prediction Bounds (Ω/Ω)	MinDS (%)
1a	[0.0–0.7]	−25 ± 3	0.88	±0.031	0.3
	[0.0–0.4]	−32 ± 6	0.86	±0.040	0.3
1b	[0.0–0.7]	−19 ± 3	0.79	±0.031	0.3
	[0.0–0.4]	−25 ± 4	0.81	±0.037	0.3
2a	[0.0–0.7]	−10 ± 5	0.30	±0.036	0.8
	[0.0–0.4]	−26 ± 10	0.55	±0.035	0.3
2b	[0.0–0.7]	−9 ± 5	0.23	±0.033	0.7
	[0.0–0.4]	−27 ± 11	0.53	±0.034	0.3
3a	[0.0–0.7]	−7 ± 3	0.39	±0.029	0.9
	[0.0–0.4]	−18 ± 3	0.83	±0.021	0.3
3b	[0.0–0.7]	−10 ± 2	0.57	±0.027	0.6
	[0.0–0.4]	−20 ± 3	0.87	±0.020	0.3

**Table 3:** Gauge factor, prediction bounds, and minimum detectable strain for each sensor estimated for the two frequencies of the dynamic test.

Sensor	Test Frequency (Hz)	GF	R2	Prediction Bounds (Ω/Ω)	MinDS (%)
1a	0.01	−27 ± 2	0.88	±0.024	0.2
	0.1	−44 ± 2	0.93	±0.026	0.2
1b	0.01	−25 ± 2	0.88	±0.023	0.2
	0.1	−38 ± 2	0.92	±0.024	0.2
2a	0.01	−43 ± 1	0.98	±0.012	0.1
	0.1	−50 ± 1	0.97	±0.016	0.1
2b	0.01	−41 ± 1	0.98	±0.012	0.1
	0.1	−51 ± 1	0.98	±0.013	0.1
3a	0.01	- ^(1)^	-	-	-
	0.1	−35 ± 1	0.97	±0.013	0.1
3b	0.01	−17 ± 1	0.81	±0.015	0.2
	0.1	−37 ± 1	0.96	±0.016	0.1

^(1)^ Results are not available due to sudden sensor failure.

## Data Availability

Data are contained within the article.
